# Determinants of health literacy in early childhood care among marginalized ethnic communities: a cross-sectional study in three border provinces of Thailand

**DOI:** 10.1186/s12939-026-02990-0

**Published:** 2026-08-21

**Authors:** Nathamon Wutthipan, Kunnara Maneekunwong, Pimkanabhorn Trakooltorwong, Srisuluk Kietmaneerat, Chuntana Reangsing, Khanittha Pitchalard, Salisa Kodyee, Katemanee Moonpanane

**Affiliations:** 1 School of Nursing, Mae Fah Luang University, Chiang Rai, Thailand; 2 Nursing Innovation Research and Resource Group, School of Nursing, Mae Fah Luang University, Chiang Rai, Thailand

**Keywords:** Health literacy, Ethnic minority, Early childhood development, Primary caregiver

## Abstract

**Background:**

Early childhood development metrics remain substandard among marginalized ethnic minority communities in three border provinces of Thailand. This study evaluated the levels and determinants of health literacy (HL) among primary caregivers to contextualize its role in mitigating adverse child health outcomes.

**Methods:**

A community-based, cross-sectional correlational study employing **multi-stage cluster random sampling** was conducted between July and October 2024. Sample size was determined using an a priori power analysis for multiple linear regression (f² = 0.05, α = 0.05, power = 0.80, 19 predictors), yielding a minimum of 351 participants; 380 caregivers were enrolled. Data were collected using the validated 61-item Parental Health Literacy Scale assessing five HL domains. Multiple linear regression with simultaneous variable entry identified independent determinants of overall HL.

**Results:**

Among the 380 participants, the largest ethnic group was Thai-Yai (23.95%), followed by Thai-Lue (19.74%), Hmong (19.21%), Akha (17.37%), Lahu (14.47%), Chinese–Yunnan (3.95%), Lee-Soo (1.05%), and Yao (0.26%). Caregivers demonstrated moderate health literacy in the functional, cognitive, critical, and behavioral domains, but exhibited low proficiency in the interactive domain (communicating care needs), resulting in a moderate overall composite score (135.04/199 = 67.9%). Multiple linear regression demonstrated that interpersonal professional support, specifically nurse-led counselling, strongly predicted health literacy proficiency (B = 9.318, *p* = 0.027). Other significant predictors included receiving information from provincial hospitals (B = 9.013, *p* = 0.030) and early childhood development centers (B = 7.531, *p* = 0.032), perceived financial adequacy (B = 3.470, *p* = 0.013), formal educational attainment (B = 3.064, *p* = 0.007), and parity (B = 3.252, *p* = 0.006), identifying primigravida caregivers as a vulnerable demographic.

**Conclusions:**

Nurse-led counselling, institutional health support from provincial hospitals and early childhood development centers, formal educational attainment, and parity are the primary modifiable determinants of HL in this population. Public health interventions must prioritize face-to-face, culturally congruent caregiver education delivered through dual-tiered institutional networks, with targeted support for first-time mothers.

## Background

Early childhood represents a critical period of neuroanatomical development, characterized by rapid synaptogenesis and high neuroplasticity across cognitive, motor, and socio-emotional domains [1]. Despite global efforts to secure developmental potential, the World Health Organization estimates that 250 million children in low- and middle-income countries (LMICs) remain at risk of failing to reach their developmental milestones [2, 3]. In Thailand, child development remains a persistent public health challenge. National longitudinal data indicate that the prevalence of developmental delays has remained stagnant over the past two decades, with rates recorded at 28.30% in 1999 and 26.60% in 2020 [4, 5]. Provincial data align with national trends: in 2025, developmental screening coverage among under 5 years was low in Chiang Rai (65.11%), Nan (76.72%), and Chiang Mai (63.75%). Among screened children, 55.22% showed appropriate development, while suspected delay ranged from 13.34 to 16.59% across provinces [6]. This mirrors a Northeastern Thailand cohort, where overall suspected delay was 22.9%, driven mainly by language (14.3%) and gross motor (10.0%) domains, with lower rates for personal-social (5.7%) and fine motor (2.9%) skills. Notably, institutionalized children showed a reversed pattern fine motor delay (50.87%) far exceeding gross motor delay (27.83%) suggesting care setting strongly shapes which developmental domains are affected [7, 8].

This lack of substantial progress indicates that clinical interventions alone are insufficient. The Lancet Early Childhood Development Series Steering Committee’s synthesis of interventions across the health, nutrition, education, child protection, and social protection sectors concluded that single-sector, clinic-based approaches consistently under-perform multi-sectoral “nurturing care” packages that equip parents and caregivers directly, and that sustainable improvement in developmental outcomes requires caregiver-directed strategies delivered alongside, not instead of, clinical services [9, 10]. Family involvement is similarly recognized as essential, functioning as an active partner and a cornerstone in ensuring the holistic well-being and development of the child [11, 12]; there is a critical need to address the socio-behavioral determinants and the disparities in health literacy that limit a caregiver’s capacity to engage in evidence-based early childhood care [13].

Health literacy (HL) the multidimensional capacity to access, understand, appraise, and apply health information is a fundamental determinant of health outcomes and a critical component of child health policy [14]. In the border provinces of Upper Northern Thailand, HL is compromised by intersecting vulnerabilities: geographic isolation, linguistic distance from the national language, and socioeconomic deprivation [15]. These barriers limit caregivers’ use of standardized tools such as the Developmental Surveillance and Promotion Manual (DSPM) [16], while the rapid, unregulated rise of screen-based media use for behavioral management often comes at the expense of language acquisition and attention span [17]. Low HL among ethnic minority caregivers thus acts as a structural barrier perpetuating intergenerational developmental inequity [18].

While HL is a recognized priority in Thailand’s 20-Year National Strategic Plan for Public Health, an implementation gap persists for marginalized border populations [19]. Current evidence on HL determinants in Thailand derives predominantly from Thai-speaking urban majority populations; the challenges of ethnic minority groups including legal precarity, statelessness, and cultural variation in caregiving remain substantially under-researched [20, 21]. A scoping review confirmed ethnic minority and border-zone populations are systematically underrepresented in the HL literature [22], and a global bibliometric analysis similarly found a scarcity of HL research in Asian low- and middle-income settings [23], underscoring the need to identify the specific factors that determine HL in these underserved communities [24]. This study aimed to analyze the levels and determinants of HL among ethnic minority caregivers in three border provinces of Northern Thailand, to provide an evidence base for culturally and linguistically inclusive public health interventions, consistent with the Sustainable Development Goal 3.8 mandate of universal health coverage and equitable child developmental outcomes [25].

## Methods

### Study design and setting

A community-based, cross-sectional correlational study identified the determinants of HL among ethnic minority primary caregivers in the border provinces of Upper Northern Thailand between July and October 2024. This region was purposively selected for its high concentration of ethnic minority groups (Thai-Yai/Shan, Thai-Lue, Hmong, Akha, and Lahu communities) and its border-zone geographic isolation and linguistic barriers.

The study population comprised primary caregivers of children under five years residing in ethnic minority communities across the border regions of three Northern Thailand provinces. Eligibility required participants to (1) be the primary caregiver of a child under five, (2) have resided in the study area for at least six months, and (3) communicate in Thai or through a trained community-based interpreter.

### Sampling and sample size

A multi-stage cluster random sampling technique was employed to ensure regional representativeness and reduce selection bias across the geographically isolated study area. In the first stage, three provinces in Upper Northern Thailand were selected via simple random sampling. In the second stage, two districts from each selected province were randomly selected, targeting areas with high-density ethnic minority settlements, resulting in six districts in total. In the final stage, systematic random sampling was applied at the village level to identify specific households.

Sample size was determined via a priori power analysis for multiple linear regression. Comparable HL studies in similar Southeast Asian ethnic minority populations report explained variance of approximately 10%-20% (f² ≈ 0.05–0.15); a conservative f² = 0.05 was specified to avoid underpowering the study if true effects were modest [26]. With α = 0.05 (two-tailed), power = 0.80, and 19 predictors, this yielded a minimum requirement of 351 participants. A 20% non-response buffer set a target of 422 caregivers; 380 completed the study.

### Research instruments

Data were collected using a structured questionnaire that underwent content validity testing by a panel of five pediatric nursing and public health experts, yielding a content validity index (CVI) > 0.80 for all items. Sociodemographic characteristics — age, formal educational attainment, ethnicity, occupation, parity, and perceived household financial status — were collected via the same questionnaire; ethnicity was self-reported from a pre-specified list of ethnic minority categories with an open-response option.

Parental health literacy was evaluated using the 61-item Parental Health Literacy Scale, adapted from Nutbeam’s multi-dimensional HL framework [14, 27], originally validated with caregivers of young children in Thai community settings [27] and adapted here with culturally appropriate language verified by two bilingual community health liaisons. Five domains were assessed across two formats: the cognitive-understanding domain (15 items) used dichotomous scoring (correct = 1, incorrect = 0; range 0–15; low < 9, moderate 9–11, high 12–15), while the four behavioral domains — access to information (15 items; range 15–60), self-management (15 items; range 15–60), decision-making (8 items; range 8–32), and communication (8 items; range 8–32) — used a 4-point Likert scale (1 = never to 4 = always), with three items per behavioral domain reverse-scored to control for acquiescence bias. Because the binary and Likert-scored domains were not directly commensurable, each domain score was transformed to a percentage of its maximum (%Max), and overall HL was expressed as the mean of the 5%Max values, with global proficiency classified as low (< 60%), moderate (60%-79%), or high (≥ 80%). The instrument showed acceptable internal consistency in a pilot cohort of 30 marginalized ethnic minority caregivers (overall Cronbach’s α = 0.75; domain-level α 0.72–0.81).

### Data collection

Ethical approval was granted by the Human Research Ethics Committee of the Chiang Rai Provincial Public Health Office (Protocol No. CRPPHO 20/2567). Research assistants, trained in cultural sensitivity and standardized data collection procedures, facilitated structured interviews. All participants provided written informed consent prior to enrolment. For caregivers with limited literacy, study information was read aloud in their native dialect by a community liaison, and consent was witnessed by a community leader in accordance with the Declaration of Helsinki.

### Data analysis

Data were analyzed using SPSS Statistics version 26 (IBM Corp., Armonk, NY, USA). Descriptive statistics summarized the demographic profile and HL levels; chi-square tests examined associations between categorical demographic variables and HL classification, and Pearson correlations assessed relationships between continuous predictors and overall HL.

Multiple linear regression with simultaneous (enter-method) variable entry was the primary analytic method. Assumptions were verified prior to regression: residual normality via the Shapiro-Wilk test and Q-Q plots; homoscedasticity via the Breusch-Pagan test; multicollinearity via variance inflation factors (VIF 1.06–2.71, all < 3.0, confirming no problematic collinearity); and independence of residuals via the Durbin-Watson statistic (d = 1.94), indicating no substantial autocorrelation. Given the multi-stage cluster random sampling design, cluster-robust standard errors were computed with district as the clustering unit, yielding an intraclass correlation coefficient (ICC) of 0.04 (DEFF = 1.12). Individual VIF values are reported in Table 4. All tests were two-tailed, with significance set at *p* < 0.05.

## Results

### Participant characteristics

Five ethnic minority groups were represented: Thai-Yai (Shan State) was largest (*n* = 91; 23.95%), followed by Thai-Lue (*n* = 75; 19.74%), Hmong (*n* = 73; 19.21%), Akha (*n* = 66; 17.37%), and Lahu (*n* = 55; 14.47%). Mean age was 30.86 years (SD = 8.50), mostly 21–30 years (48.16%). Most participants were farmers (62.63%) or general laborers (32.11%); over half had secondary education (50.79%). Monthly income was predominantly below 10,000 baht (42.63% < 5,000; 42.37% 5,000–10,000), and 38.16% reported sufficient income with debt versus 25.53% with insufficient income and debt. Nearly half (41.58%) were experiencing their first pregnancy, and 96.84% reported no history of genetic diseases (Table 1).

**Table 1:** Socio-demographic information (*N* = 380)

Characteristics	*n*	%
Ethnicity		
Thai-Yai (Shan state)	91	23.95
Thai-Lue	75	19.74
Hmong	73	19.21
Akha	66	17.37
Lahu	55	14.47
Chinese Yunann	15	3.95
Lee-Soo	4	1.05
Yao	1	0.26
Province		
Chiang Rai	234	15.00
Chiang Mai	57	62.00
Nan	89	23.00
Age (years) (Mean = 30.86, SD = 8.50)		
< 20	19	5.00
21–30	183	48.16
31–40	133	35.00
41–50	34	8.95
≥ 51	11	2.90
Mother (365, 96.05%; Father 15; 3.95%)		
Formal Educational Attainment		
No formal education	53	13.95
Primary education	112	29.47
Secondary education	193	50.79
Diploma or higher	22	5.79
Socio-Economic Profile		
Occupation		
Farmer	238	62.63
General laborer	122	32.11
Vendor	20	5.26
Average Monthly Income (THB)		
< 5,000	162	42.63
5,000–10,000	161	42.37
10,000–15,000	40	10.53
≥ 15,000	17	4.47
Obstetric & Health History		
First pregnancy (Primigravida)	158	41.58
Second pregnancy	116	30.54
≥ Third pregnancy	106	27.89
History of Genetic Diseases		
No	368	96.84
Yes	12	3.16

**Table 2:** Utilization of health information (*N* = 380)

	*n* (Yes)	%
Informal & Community Support Networks		
Friends	139	36.58
Parents	114	30.00
Early childhood development centers	80	21.05
Institutional & Professional Health Services		
Sub-district health promoting hospitals	168	44.21
Nurse-led counselling	54	14.21
Provincial hospitals	46	12.11
Private Clinics	36	9.47
Printed Health Materials		
MCH	159	41.84
DSPM	41	10.79
Digital & Broadcast Media Exposure		
Online media	120	31.58
Television	35	9.21
Village public broadcasting	11	2.89

Note: Percentages do not total 100% as participants could select multiple sources of information

**Table 3:** Health literacy proficiency domains (*N* = 380)

Health Literacy Domains	Actual Range	Mean (SD)	95% CI Lower	95% CI Upper	Interpretation
1. Functional Domain: Accessing health information	15–60	39.28 (11.27)	38.15	40.42	Moderate
2. Cognitive Domain: Understanding early childhood care	5–15	11.09 (1.69)	10.92	11.26	Moderate
3. Critical Domain: Appraisal and decision-making	8–32	19.94 (6.12)	19.33	20.56	Moderate
4. Behavioral Domain: Self-management practices	29–60	44.82 (7.61)	44.06	45.59	Moderate
5. Interactive Domain: Communicating care needs	8–32	18.89 (5.94)	18.29	19.49	Low
Overall Health Literacy score	72–192	135.04 (24.82)	132.54	137.55	Moderate

**Table 4:** Structural and interpersonal determinants of health literacy

Variable	B (Adj.)	SE	Beta	t	Crude B 95% CI	Crude Sig.	Adjusted 95% CI	Adjusted Sig.
Constant	107.691	8.750	—	12.308	—	—	90.481, 124.901	< 0.001
*Socio-Demographic & Biological Determinants*								
Age	-0.055	0.161	-0.018	-0.343	-0.132, 0.457	0.279	-0.373, 0.262	0.732
Experiential Health Knowledge (Parity)	3.252	1.180	0.148	2.755	1.009, 5.371	0.004**	0.930, 5.574	0.006**
Family Health History	6.448	7.601	0.045	0.848	-9.557, 19.107	0.513	-8.504, 21.400	0.397
*Socio-Economic Determinants*								
Formal Educational Attainment	3.064	1.131	0.149	2.708	2.537, 6.529	< 0.001**	0.838, 5.289	0.007**
Occupation	1.331	1.187	0.059	1.121	0.175, 4.673	0.105	-1.005, 3.666	0.263
Household Income	-0.491	0.717	-0.037	-0.685	-1.770, 0.905	0.525	-1.900, 0.919	0.494
Perceived Financial Adequacy	3.470	1.384	0.141	2.507	-0.057, 4.922	0.055	0.747, 6.193	0.013*
*Social & Household Environment*								
Household Structure	10.950	9.205	0.061	1.190	0.914, 38.005	0.040*	-7.156, 29.057	0.235
Informal Social Support (Parents)	-1.150	3.039	-0.021	-0.378	-4.039, 6.902	0.607	-7.128, 4.826	0.705
Informal Social Support (Friends)	-0.812	2.925	-0.016	-0.278	-1.105, 9.276	0.123	-6.564, 4.940	0.781
*Health Information Received From*								
Nurse-Led Counselling	9.318	4.186	0.132	2.226	7.469, 21.531	< 0.001**	1.084, 17.553	0.027*
Child Development Centers	7.531	3.499	0.123	2.152	6.014, 18.073	< 0.001**	0.649, 14.412	0.032*
Sub-District Health Promoting Hospitals	-5.085	3.720	-0.074	-1.367	-12.568, 0.873	0.088	-12.401, 2.231	0.173
Private Clinic	3.391	4.602	0.040	0.737	3.169, 20.355	< 0.001**	-5.660, 12.441	0.462
Provincial Hospitals	9.013	4.135	0.116	2.180	4.202, 19.543	0.003**	0.881, 17.145	0.030*
*Media & Digital Material / Channel*								
Online Platform	5.183	3.154	0.096	1.643	4.600, 15.203	< 0.001**	-1.020, 11.386	0.101
Broadcasting Television	7.163	4.820	0.083	1.486	6.041, 23.133	< 0.001**	-2.319, 16.642	0.138
MCH Handbook	2.005	2.773	0.040	0.723	3.009, 13.046	0.002**	-3.448, 7.458	0.470
DSPM	-4.112	4.599	-0.050	-0.894	-4.321, 11.781	0.363	-13.156, 4.932	0.372

Note(s): *Statistically significant at *p* ≤ 0.05, **p < 0.01; constant = 107.691, R² = 0.197, adjusted R² = 0.145, F(22, 339) = 3.787, *p* < 0.001

### Health information sources

Caregivers reported diverse health-information sources. The most cited institutional source was sub-district health promoting hospitals (*n* = 168; 44.21%), followed by the Maternal and Child Health (MCH) handbook (*n* = 159; 41.84%). Informal sources friends (36.58%) and parents (30.00%) were also prominent, along with online media (31.58%); nurse-led counselling (14.21%) and provincial hospitals (12.11%) were less common (Table 2).

### Health literacy levels

HL proficiency across five domains was moderate for four (functional 65.5%; cognitive 73.9%; critical 62.3%; behavioral 74.7%) and low for the interactive domain (59.0%, below the < 60% threshold). The overall composite score (135.04/199 = 67.9%) was moderate, per the study’s 60%-79% moderate band (Table 3).

### Determinants of health literacy

Multiple linear regression identified six statistically significant independent predictors of overall HL (Table 4). The model was statistically significant (F(22, 339) = 3.787, *p* < 0.001) and explained 14.5% of the variance in HL (adjusted R² = 0.145). Professional support via nurse-led counselling was the strongest predictor (B = 9.318, 95% CI 1.084 to 17.553, *p* = 0.027), followed by provincial hospitals (B = 9.013, 95% CI 0.881 to 17.145, *p* = 0.030) and child development centers (B = 7.531, 95% CI 0.649 to 14.412, *p* = 0.032). Perceived financial adequacy (B = 3.470, 95% CI 0.747 to 6.193, *p* = 0.013), experiential health knowledge through parity (B = 3.252, 95% CI 0.930 to 5.574, *p* = 0.006), and formal educational attainment (B = 3.064, 95% CI 0.840 to 5.289, *p* = 0.007) were also significant, with none of their 95% CIs crossing zero. The remaining thirteen predictors did not reach significance, with 95% CIs spanning zero (Table 4).

## Discussion

This study evaluated HL levels and their determinants among primary caregivers in marginalized ethnic minority communities in Thailand’s highland border provinces. The overall composite HL score (67.9%) was moderate per the study’s thresholds (60%-79% = moderate). The interactive communication domain (59.0%) was the only domain classified as low; the critical appraisal domain (62.3%), the second weakest, remains a priority for intervention despite its moderate classification. These findings indicate that while caregivers can access and functionally comprehend basic health information, they show low capacity to communicate care needs within the health system and limited capacity to critically evaluate health information. In culturally and linguistically diverse border areas, structural educational disparities often limit HL to basic functional compliance rather than critical empowerment [28, 29], placing these caregivers and their children at a pronounced disadvantage. Although child developmental or morbidity outcomes were not directly assessed, caregiver health literacy deficits hold clear implications for pediatric health. A systematic review demonstrated consistent associations between caregiver health literacy and children’s health knowledge, behaviors, service utilization, and morbidity, with the strongest associations observed in provider communication and critical appraisal domains identified as weakest in this cohort [30]. The moderate-to-low health literacy proficiency documented, particularly the diminished interactive domain, suggests potential vulnerability to suboptimal child outcomes, warranting future research integrating caregiver health literacy with measured pediatric outcomes.

Nurse-led counselling was the strongest independent predictor of caregiver HL (B = 9.318, *p* = 0.027), consistent with conceptual models linking face-to-face health communication to improved HL through enhanced access, understanding, and navigation [31]. Evidence across Asian countries similarly shows that culturally congruent, interpersonal communication is the most effective modality for health-knowledge transfer among ethnic minority and rural populations [24], paralleling community health worker programs in Indonesia [32] and maternal health campaigns in Vietnam [33, 34]. This interpretation must be qualified by the cross-sectional design, which precludes establishing directionality: caregivers with higher baseline HL may be more likely to seek nurse-led counselling, rather than counselling producing the HL gain [35]. Longitudinal or interventional designs are required before this association can be interpreted as causal.

Beyond professional support, structural socioeconomic factors were significant predictors. Formal educational attainment (B = 3.064, *p* = 0.007) and perceived income adequacy (B = 3.470, *p* = 0.013) were significant, whereas objective monthly household income was not (B = − 0.491, *p* = 0.494). This dissociation is consistent with relative deprivation theory, which posits that perceived sufficiency, rather than absolute income, shapes health behaviors and information-seeking capacity [36]. Where the majority earn below 10,000 THB per month, the absolute income differential is narrow; perceived capacity to manage basic needs better differentiates HL levels. These findings contextualize the socio-ecological risk profile for developmental delay documented across Southeast Asia [37, 38], paralleling marginalized highland populations in Laos and Myanmar, where restricted access to routine developmental surveillance limits caregiving capacity [39], underscoring HL as a mediating pathway between economic marginalization and poor child health outcomes [40].

Experiential health knowledge through parity was also significant (B = 3.252, *p* = 0.006): multiparous caregivers accrue longitudinal exposure to health information that facilitates the transition from theoretical knowledge to practical competence, leaving first-pregnancy mothers a vulnerable subgroup requiring targeted, proactive support [41]. This also contextualizes the non-significant effect of informal social support from friends and parents (both *p* > 0.70), suggesting peer-transmitted knowledge is insufficiently structured to translate into measurable HL gains. Although 31.58% of caregivers used online media, this did not reach significance as an HL predictor (B = 5.183, *p* = 0.101); the critical-domain deficit (62.3%) observed here is consistent with findings from Southeast Asian populations where digital access has outpaced critical evaluation skills [42, 26], leaving caregivers at risk of adopting unverified, digitally sourced practices. Interpretation of these behavioral and interactive findings should account for reliance on self-reported measures, which are subject to recall error and social desirability bias and may understate the true magnitude of the deficits identified [43].

The institutional source of health information also predicted HL. Caregivers supported by child development centers (B = 7.531, *p* = 0.032) and provincial hospitals (B = 9.013, *p* = 0.030) had significantly higher HL scores, whereas sub-district health-promoting hospitals showed a non-significant, negative association (B = − 5.085, *p* = 0.173). This divergence may reflect structural differences in service delivery: sub-district hospitals function primarily as passive, screening-oriented access points with limited structured HL education, whereas provincial hospitals and child development centers engage caregivers in more sustained, information-rich interactions. The multi-stage cluster random sampling strategy ensured regional representativeness across geographically isolated communities and reduced selection bias.

This study’s methodological strengths reinforce confidence in the findings. The scale showed strong internal consistency, and the percentage-of-maximum aggregation approach addressed the psychometric incommensurability between binary- and Likert-scaled domains. Its multidimensional structure five distinct HL domains rather than a single composite construct distinguished the interactive and critical-appraisal deficits from comparatively stronger domains, a pattern a unidimensional measure would likely obscure. This is underscored by comparison with the DSPM, Thailand’s surveillance tool, which was similarly non-significant (B = − 4.112, *p* = 0.372), possibly reflecting literacy barriers to its use.

Several limitations warrant consideration. The model explained only 14.5% of adjusted variance, leaving substantial variability unaccounted for by the 19 predictors examined. Plausible unmeasured constructs—Thai language proficiency, acculturation, caregiver mental health, geographic distance to care, and household social capital—were not captured; evidence from other linguistic-minority populations suggests these independently predict health literacy beyond standard socio-demographic covariates [44], so the determinants identified here represent a partial, not complete, account. Cluster-robust standard errors and ICC estimation (ICC = 0.04, DEFF = 1.12) addressed the clustered sampling design, though formal multilevel modeling remains an important refinement for future work [45]. Because this study was purposively conducted in Northern Thailand’s highland border provinces, generalizability to urban, non-minority, or other ASEAN ethnic-minority populations should not be assumed. Finally, caregiver HL was measured as the primary outcome; children’s developmental or health outcomes were not directly assessed, and prospective studies linking caregiver HL to measured child developmental indices are needed to confirm this pathway empirically. To address persistent gaps in early childhood development among Thailand’s border communities, policy should align with Thailand’s 20-Year National Strategic Plan [19] and the ASEAN Post-2015 Health Development Agenda [46, 47], extending beyond passive screening toward nurse-led outreach that strengthens caregivers’ interactive and critical-appraisal HL skills.

## Conclusions

Nurse-led counselling, institutional health support from provincial hospitals and community child development centers, formal educational attainment, and experiential health knowledge through parity are the primary independent determinants of early childhood care HL among ethnic minority primary caregivers in Thailand’s border provinces. The overall HL composite score was classified as moderate (67.9%); the interactive communication domain alone was classified as low (59.0%). The remaining four domains were also moderate. Deficits in interactive communication and critical health information appraisal represent the most actionable areas for intervention. To improve early childhood development metrics in highland border regions, public health policy must transition from passive developmental screening to direct, culturally congruent, face-to-face health education. Regional health systems should leverage dual-tiered institutional networks and provide targeted support for first-time (Primigravida) mothers. The role of the sub-district health-promoting hospital tier and the DSPM in ethnic minority communities warrants re-evaluation in future research. Because caregiver health literacy is a recognized upstream determinant of child health knowledge, service utilization, and morbidity, strengthening it through these channels represents a modifiable pathway toward improved early childhood health outcomes in this population, a pathway that prospective research directly linking caregiver HL to measured child outcomes should confirm.

## Data Availability

No datasets were generated or analysed during the current study.

## Abbreviations

ASEAN: Association of Southeast Asian Nations
CVI: Content Validity Index
DEFF: Design Effect
DSPM: Developmental Surveillance and Promotion Manual
HL: Health Literacy
ICC: Intraclass Correlation Coefficient
LMICs: Low- and Middle-Income Countries
MCAR: Missing Completely at Random
MCH: Maternal and Child Health
SD: Standard Deviation
SDG: Sustainable Development Goal
SE: Standard Error
SPSS: Statistical Package for the Social Sciences
STROBE: Strengthening the Reporting of Observational Studies in Epidemiology
THB: Thai Baht
VIF: Variance Inflation Factor
